# Editorial: World Hepatitis Day 2024: advancing hepatitis elimination, public health strategies and innovations

**DOI:** 10.3389/fpubh.2025.1765352

**Published:** 2026-01-07

**Authors:** Arturo Panduro, Sonia Roman

**Affiliations:** 1 Department of Genomic Medicine in Hepatology, Civil Hospital of Guadalajara, Guadalajara, Jalisco, Mexico; 2 Health Sciences Center, University of Guadalajara, Guadalajara, Jalisco, Mexico

**Keywords:** antiviral agents, blood safety, disease elimination, epidemiology, health equity, public health, vaccination, viral hepatitis

## Overview

Viral hepatitis remains as major global health challenge, causing over one million deaths annually. Although hepatitis B vaccination and hepatitis C treatment have improved outcomes, efforts to eliminate the disease remain inconsistent across regions. The World Health Organization (WHO) has set a goal to eliminate viral hepatitis as a public health threat by 2030 (1). However, the path to this goal is complicated by new and overlapping causes of liver injury, such as metabolic dysfunction-associated steatotic liver disease (MASLD) (2) and sepsis-related liver injury (SRLI) (3), which can accelerate disease progression and complicate diagnosis. These overlapping factors emphasize the importance of integrated liver health strategies that combine prevention, awareness, metabolic risk reduction, and better diagnostics to achieve elimination targets.

The Research Topic *World Hepatitis Day 2024: Advancing Hepatitis Elimination, Public Health Strategies and Innovations* provides a timely opportunity to assess global efforts, identify ongoing gaps, and build momentum toward the goal set by the WHO. Sadly, the burden of hepatitis disproportionately affects low- and middle-income countries, where limited resources, weaker health systems, and socioeconomic inequalities restrict access to prevention, screening, and treatment. Stigma, migration patterns, and social determinants of health further complicate epidemiological trends and hinder engagement with care. This Research Topic includes original articles, meta-analyses, study protocols, and viewpoints from various regions and disciplines, all of which focus on a central theme: the urgent need to translate epidemiological findings, preventive strategies, and innovative tools into tangible progress toward the eradication of hepatitis. We invite our readers to explore this compilation, which covers epidemiology, awareness, immune/vaccination responses, innovations, and regional experiences, all of which are aligned with the WHO's elimination goals.

## Epidemiological and awareness insights

Studies conducted by Ding et al. in Wuxi City, in eastern China, tracked the spatial–temporal evolution of hepatitis C. These revealed that the virus is increasing and becoming more prevalent in specific geographic areas. However, elimination efforts are still fragmented and lack targeting. Without enhanced surveillance, tailored interventions, and integration into broader public health strategies, Wuxi risks failing to meet the 2030 elimination goal. In Beijing, Li et al. conducted a local case study using epidemiological models (age, period, and cohort) to identify risk gaps and guide targeted measures. Their findings show a decline in chronic hepatitis B incidence from 2005 to 2022, but they also reveal gaps in vaccination, diagnosis, and treatment, particularly among specific age and gender groups. Additionally, the cross-sectional study by Dai et al. in Anhui Province, China, highlighted a gap between knowledge and behavior. While many individuals know the basic transmission routes, misconceptions and risky practices remain widespread. Strengthening community-level education, targeted awareness campaigns, and access to direct-acting antivirals is essential to meeting the WHO's 2030 elimination target.

Meanwhile, Kremer-Flach et al.'s workbook-based method aimed to establish a baseline for national elimination strategies in Germany. However, studies by Moser et al. found that low uptake, inconsistent billing, and poor data quality in Germany's “Check-Up 5” program (BeoNet-Halle database) limited the effectiveness of hepatitis B and C screening and hindered proper evaluation.

In the Netherlands, Generaal et al. identified a significant gap in the Dutch public health system; despite high disease rates and national guidelines, migrants from endemic countries were not routinely screened for hepatitis B, hepatitis C, or HIV. This left many infections undiagnosed and untreated. These efforts reflect a broader recognition that elimination requires integrating hepatitis testing into routine health systems and reaching populations often left behind.

In other regions of the world, four studies from Ethiopia revealed the risk of acquiring and transmitting viral hepatitis among vulnerable groups, shedding light on the often-overlooked burden of these infections. Abdi et al. identified an intermediate HBsAg prevalence among diabetic patients in eastern Ethiopia and recommended routine screening, expanded vaccination coverage, and education to reduce HBV transmission. Tsegaye Amlak et al. conducted an awareness survey among barbers in East Gojjam, northwest Ethiopia, which uncovered unsafe hepatitis B prevention practices, including low education levels, poor knowledge, lack of UV sterilizer use, and negative attitudes. Tsehaynew et al. examined public hospitals in Addis Ababa and reported that informal caregivers had lower awareness of and adherence to prevention practices compared to national averages. Data from a survey by Betela et al. documented HCV prevalence and occupational risk among medical waste handlers in Ethiopia's Sidama region, showing a higher infection rate than in the general population, thus filling a gap in national surveillance. Urgent efforts are needed to enhance prevention and develop policies to prevent the spread of the hepatitis C virus.

## Immune/vaccination response and blood safety

In this section, two studies used the U.S. NHANES database, which combines clinical, laboratory, and demographic data. This enables strong population-level evidence and long-term follow-up. Hu et al. identified a significant association between a low antibody response after hepatitis B vaccination and systemic immune-inflammatory indices (SII), which predict vaccine efficacy. Meanwhile, Qiu et al. examined the neutrophil-to-lymphocyte ratio (NLR) to explore how systemic inflammation and immune activation relate to the risk of all-cause mortality in individuals with resolved HBV infection. These studies shift the attention from viral persistence to systemic inflammation and metabolic comorbidities as factors affecting long-term outcomes in HBV survivors. Both articles emphasize the usefulness of simple, blood-derived biomarkers, such as the NLR and SII, as indicators of disease risk or immune performance.

Wang et al. conducted clinical studies on the duration of the immune response after HBsAg clearance with PegIFNα-2b, highlighting the complexities of long-term management connecting prevention and treatment. In addition, Sun et al.'s pharmacovigilance research, supported by the Vaccine Adverse Event Reporting System (VAERS), demonstrated the value of safety monitoring through the registration of Events Supposedly Attributable to Vaccination or Immunization (ESAVI).

Finally, the review by Huang, He et al. of transfusion-transmitted occult HBV infection reminded us that the disease remains undetected by current blood donor screening protocols, which creates a residual risk of transfusion-transmitted HBV infection and challenges global blood safety.

## Innovative strategies toward elimination

Liu et al. developed a validated predictive model to evaluate the risk of neonatal HBV infection from infected mothers and to guide prophylactic measures for their infants. The study integrates epidemiology, diagnosis, and treatment, allowing for early, personalized preventive strategies, an important innovation in the fight against vertical HBV transmission.

Huang, Feng et al. created a new model by systematically evaluating six widely used risk scores (REACH-B, GAG-HCC, CU-HCC, PAGE-B, mPAGE-B, and CAMD) to assess their ability to predict hepatocellular carcinoma risk in patients with chronic hepatitis B. However, their real-world applicability is limited by geographic and patient characteristics. This underscores the need for more refined, region-specific models to improve early detection and support the WHO's 2030 elimination goals.

Zhou et al.'s interferon therapy protocol for chronic hepatitis B patients with MAFLD showed that elimination is not straightforward. This protocol aimed to examine how metabolic dysfunction affects immune clearance and treatment efficacy, thereby revealing unresolved links between viral hepatitis and comorbidities. The authors emphasize the necessity of integrated solutions that incorporate metabolic and immunological insights into hepatitis elimination initiatives. This pioneering clinical and translational investigation illustrates the obstacles and prospects for eliminating global hepatitis.

While viral hepatitis B and C are the leading causes of chronic liver disease, recent research highlighted SRLI as an important differential diagnosis. SRLI often occurs in critically ill patients and can closely mimic viral hepatitis. He et al. have broadened the scope of diagnosing acute hepatic dysfunction by developing a new prediction model that uses routine serum markers to help clinicians distinguish SRLI from other hepatic dysfunctions and intervene promptly, ultimately reducing mortality rates among septic patients.

## Regional policies and solutions

Shindano and Horsmans emphasized the urgent need for a national policy in the Democratic Republic of the Congo to protect healthcare workers (HCWs) from hepatitis B. Despite global goals, HCW vaccination rates remain low, hindered by poor training, a lack of protective gear, and an absence of systematic accident management. Their article highlights HCWs as high-risk subjects and potential sources of transmission, shifting responsibility from individual compliance to systemic accountability. The authors propose three levels of prevention: individual precautions, institutional training and vaccination, and national policies for routine immunization and monitoring. Making vaccination mandatory for HCWs and students, with training costs covered, is a practical step toward global hepatitis elimination.

Finally, the REVIRAL project, led by Crespo et al., offered a tailored plan to eliminate viral hepatitis in Latin America. This plan is aligned with the WHO's 2030 goals through a collaborative strategy. It features a three-phase model: evaluating epidemiology and health systems, implementing universal screening and micro-elimination, and continuous monitoring with a global indicator. To overcome barriers such as fragmented services, limited diagnostics and treatments, weak referral systems, stigma, and low vaccination rates, the project includes strategies such as developing national plans, ensuring equitable access to antivirals, targeting high-risk groups, launching awareness campaigns, simplifying diagnostics, and integrating with HIV and syphilis programs.

## Challenges and future directions

Despite progress worldwide, the fight against hepatitis faces ongoing, region-specific challenges. In China, hepatitis elimination strategies are fragmented and unfocused, increasing the risk of non-compliance with WHO targets. Germany's screening programs suffer from low participation and poor data quality, while the Netherlands faces undiagnosed infections among migrants due to gaps in routine screening. In Ethiopia, vulnerable groups such as diabetics, barbers, informal caregivers, and medical waste handlers remain at higher risk due to a lack of awareness and unsafe practices. In Latin America, efforts are hampered by fragmented healthcare systems, unequal access to diagnostics and treatment, weak referral and follow-up systems, and low vaccination uptake. Similarly, the Democratic Republic of the Congo's experience underscores the need for accountability and policy change, demonstrated by mandatory hepatitis B vaccination for healthcare workers and students, supported by institutional training and national policies. Stigma and limited awareness further hinder prevention and care.

Globally, gaps in vaccination, diagnosis, and treatment endure, with blood safety threatened by undetected transfusion-transmitted hepatitis B. Innovative predictive models often fall short because they aren't tailored to local contexts, which limits their usefulness. New issues are emerging: MASLD is quickly becoming the most prevalent chronic liver condition, often overlapping with viral hepatitis and speeding disease progression. Meanwhile, SRLI complicates diagnosis in critically ill patients, as its symptoms resemble those of viral hepatitis. Addressing these overlapping issues calls for integrated liver health strategies that combine prevention, metabolic risk reduction, and better diagnostics.

Looking ahead, eliminating hepatitis will require unified, adaptable, and collaborative strategies. Assessing epidemiology and health system capacity, implementing universal screening and micro-elimination programs, and maintaining ongoing monitoring with global indicators offer valuable lessons for every region. Regardless of the local context, success depends on strong national planning, equitable access to diagnostics and treatment, targeted interventions for high-risk groups, and the integration of hepatitis efforts with other public health initiatives. By fostering regional cooperation, simplifying care pathways, and launching widespread awareness campaigns, countries can break down barriers such as fragmented health systems, limited resources, and social stigma. Ultimately, a comprehensive, flexible, and inclusive approach is essential for all regions to accelerate progress and achieve the WHO's 2030 hepatitis elimination goals.
